# Supplementary material for: Predicting visual function by interpreting a neuronal wiring diagram
Source: Nature. 2024 Oct 2;634(8032):113–23. doi: 10.1038/s41586-024-07953-5 (PMC11446822; doi:10.1038/s41586-024-07953-5)
Supplement: Supplementary file 6 — CRF and ERF predictions for individual TmY4 and TmY9 cells. Analogous to Supplementary Data 3, but for TmY target types. Shown are the top four monosynaptic pathways, the strongest pathway passing through each of the top ten intermediary types (ranking from Extended Data Fig. 7), and the trisynaptic pathway Tm1–TmY–Dm3–TmY (see the section entitled Prediction of spatial normalization). [file 41586_2024_7953_MOESM6_ESM.zip › DataS4/TmY9q/720575940619873974.pdf]

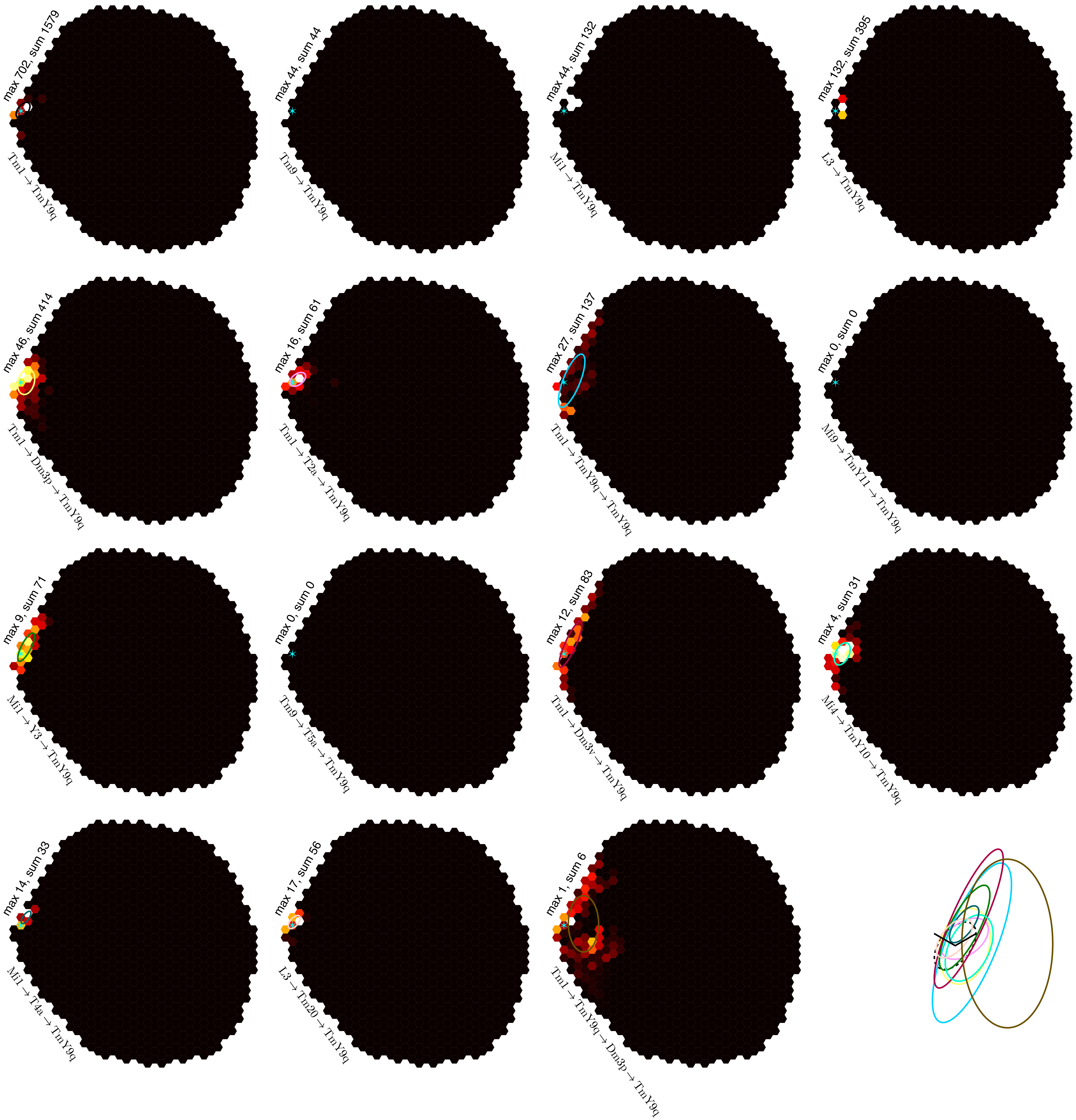

max 702, sum 1579
Tm1 → TmY9q
max 44, sum 44
Tm9 → TmY9q
max 44, sum 132
Mi1 → TmY9q
max 132, sum 395
L3 → TmY9q
max 46, sum 414
Tm1 → Dm3p → TmY9q
max 16, sum 61
Tm1 → T2a → TmY9q
max 27, sum 137
Tm1 → TmY9q → TmY9q
max 0, sum 0
Mi9 → TmY11 → TmY9q
max 9, sum 71
Mi1 → Y3 → TmY9q
max 0, sum 0
Tm9 → T5a → TmY9q
max 12, sum 83
Tm1 → Dm3v → TmY9q
max 4, sum 31
Mi4 → TmY10 → TmY9q
max 14, sum 33
Mi1 → T4a → TmY9q
max 17, sum 56
L3 → Tm20 → TmY9q
max 1, sum 6
Tm1 → TmY9q → Dm3p → TmY9q
